# Transcriptome-Wide Identification of WRKY Transcription Factor and Functional Characterization of *RgWRKY37* Involved in Acteoside Biosynthesis in *Rehmannia glutinosa*

**DOI:** 10.3389/fpls.2021.739853

**Published:** 2021-09-29

**Authors:** Fengqing Wang, Xinrong Li, Xin Zuo, Mingming Li, Chunyan Miao, Jingyu Zhi, Yajing Li, Xu Yang, Xiangyang Liu, Caixia Xie

**Affiliations:** ^1^College of Agronomy, Henan Agricultural University, Zhengzhou, China; ^2^School of Medicine, Henan University of Chinese Medicine, Zhengzhou, China

**Keywords:** acteoside biosynthesis, WRKY transcription factor, transcriptome, expression analysis, *Rehmannia glutinosa*

## Abstract

WRKYs play important roles in plant metabolism, but their regulation mechanism in *Rehmannia glutinosa* remains elusive. In this study, 37 putative WRKY transcription factors (TFs) with complete WRKY domain from *R. glutinosa* transcriptome sequence data were identified. Based on their conserved domains and zinc finger motif, the *R. glutinosa* WRKY TFs were divided into five groups. Structural feature analysis shows that the 37 RgWRKY proteins contain WRKYGQK/GKK domains and a C2H2/C2HC-type zinc finger structure. To identify the function of *RgWRKY* members involved in acteoside biosynthesis, transcriptional profiles of 37 *RgWRKYs* in hairy roots under salicylic acid (SA), methyl jasmonate (MeJA), and hydrogen peroxide (H_2_O_2_) treatments were systematically established using RNA-seq analysis. Based on the correlationship between the expression levels of *RgWRKY* genes and acteoside content, *RgWRKY7*, *RgWRKY23*, *RgWRKY34*, *RgWRKY35*, and *RgWRKY37* were suggested to be involved in acteoside biosynthesis in *R. glutinosa*, and *RgWRKY37* was selected for gene functional research. Overexpression of *RgWRKY37* increased the content of acteoside and total phenylethanoid glycosides (PhGs) in hairy roots and enhanced the transcript abundance of seven enzyme genes involved in the acteoside biosynthesis pathway. These results strongly suggest the involvement of the WRKY transcription factor in the regulation of acteoside biosynthesis.

## Introduction

*Rehmannia glutinosa* L. is a perennial herb of Scrophulariaceae and is widely distributed in Northern China, including Henan and Shanxi provinces. Many tissues and organs of *R. glutinosa* plants, especially tuberous roots and leaves, are rich in many active components, such as iridoid glycosides (IGs), phenylethanoid glycosides (PhGs), flavonoids, polysaccharides, and amino acids (Zhang et al., 2008a; Liu et al., 2014). The tuberous root of *R. glutinosa* is a traditional Chinese herb, recorded in the Chinese medical classics as “Shennong’s Herbal.” It is considered a “top-grade” herb in China (Zhi et al., 2018). Many studies have revealed that the tuberous root of *R. glutinosa* is rich in a variety of active ingredients with pharmacological activity in the human immune, the blood system, and the endocrine, cardiovascular, and nervous system (Zhang et al., 2008b). *Rehmannia glutinosa* has become an effective traditional Chinese medicine (TCM) for decreasing blood glucose (Qin et al., 2018), and it has also been noted for its antioxidant (Li et al., 2020a), anti-inflammatory (Liu et al., 2012), anti-depressant (Wang et al., 2018), and anti-aging (Bai et al., 2018) properties.

Acteoside, originally isolated from *Verbascum sinuatum* L. (Scarpati and Monache, 1963), is a naturally occurring component in various plants, such as Scrophulariaceae, Verbenaceae, and Oleaceae. Modern pharmacological studies have shown that acteoside has biological activities, such as antioxidant, anti-inflammatory, antinephritic, hepatoprotective, immunomodulatory, and neuroprotective (He et al., 2011). Acteoside has a high content in the leaves and tuberous roots of *R. glutinosa*, and this ingredient is often used as one of the quality-control components of *R. glutinosa*. Acteoside belongs to the PhG compounds and consists of four moieties: caffeic acid (CA), hydroxytyrosol (3,4-dihydroxyphenylethanol), glucose, and rhamnose (Saimaru and Orihara 2010). Current research results report that acteoside is synthesized from caffeoyl CoA *via* the phenylalanine pathway and hydroxytyrosol glucoside *via* the tyrosine-derived pathway (Supplementary Figure S1; Wang et al., 2017a; Zhou et al., 2020). Based on metabolomic analysis and transcriptome sequencing, key intermediates in the acteoside biosynthesis pathway were identified, and its enzymes and their corresponding encoding genes (Saimaru and Orihara, 2010; Zhou et al., 2016; Wang et al., 2017a; Zhi et al., 2018). However, the molecular regulation of transcription factors in acteoside biosynthesis of *R. glutinosa* is still unknown.

Transcription factors (TFs) play a crucial role in plants by controlling the expression of genes involved in various cellular processes (Han et al., 2014; Chen et al., 2018). The WRKY gene family is the seventh largest TFs family in flowering plants and contain about 60 amino acid long four-stranded β-sheet WRKY DNA-binding domains and a zinc finger motif (Rushton et al., 2010). WRKY proteins are divided into three groups (I, II, and III) based on the type of zinc finger motif and the number of WRKY domains (Eulgem et al., 2000). Many WRKY genes have been identified from different plant species and reported to be involved in or respond to plant development, metabolism, and various biological processes such as biotic and abiotic stresses (Jiang et al., 2016; Chen et al., 2018, 2019). In recent years, there has been an increasing number of studies on the regulation of WRKY transcription factors on the accumulation and change in secondary metabolites in medicinal plants. For example, *CrWRKY1* overexpressed in *Catharanthus roseus* hairy roots is involved in indole alkaloid biosynthetic by binding to the promoter of the d tryptophan decarboxylase (TDC) gene, increasing serpentine levels up to 3-fold (Suttipanta et al., 2011). Overexpression of *SmWRKY1* significantly increased the expression levels of genes encoding 1-deoxy-D-xylulose-5-phosphate synthase (DXS) and 1-deoxy-D-xylulose-5-phosphate reductoisomerase (DXR), resulting in a more than 5-fold increase in tanshinone production in transgenic lines of *Salvia miltiorrhiza* (Cao et al., 2018). In *Ophiorrhiza pumila*, *OpWRKY2* positively regulates camptothecin biosynthesis by binding to and activating the camptothecin pathway gene *OpTDC* (Hao et al., 2021), but *OpWRKY1* negatively regulates camptothecin biosynthesis by directly downregulating the transcription of P450 reductase (*CPR*) coding gene (Xu et al., 2020). Therefore, further studies are needed on the function of WRKY genes in regulating phenylethanol glycoside biosynthesis in plants.

In this study, a transcriptome-wide survey and systematic characterization of the WRKY family in *R. glutinosa* were carried out. A phylogenetic tree was constructed combining WRKY proteins from *R*. *glutinosa*, *Arabidopsis thaliana*, and *Oryza sativa* was constructed to test their evolutionary relationships. The WRKY gene involved in acteoside biosynthesis was investigated using salicylic acid (SA), methyl jasmonate (MeJA), and H_2_O_2_ to induce a response expression profile in *R. glutinosa* hairy roots. Further, the expression patterns of WRKY genes in different tissues of *R. glutinosa* were detected by RNA-seq and quantitative real-time PCR (qRT-PCR). We identified five candidate WRKY genes potentially involved in the regulation of the acteoside biosynthesis. Furthermore, in the functional characterization, overexpression of *RgWRKY37* significantly increased the content of acteoside in *R. glutinosa* transgenic hairy roots. Our results suggest that WRKY genes play important roles in acteoside biosynthesis.

## Materials and Methods

### Plant Materials

#### Plant Material and Sample Collection

*Rehmannia glutinosa* hairy roots were generated by *Agrobacterium rhizogenes*-mediated transformation of W85-5 leaves, as previously described by Wang et al. (2017a). After SA (25μmol/L) and MeJA (5μmol/L) treatments for 0, 12, and 24h, hairy roots were collected for RNA-seq analysis. qRT-PCR analysis was performed on hairy roots treated with SA and MeJA for 3, 9, 12, and 24h. After treatment with H_2_O_2_ (1mmol/L) for 0, 12, 24, and 36h, hairy roots were collected for RNA-seq and qRT-PCR analysis. The tuberous roots of the *R. glutinosa* cultivar W85-5 were planted at the planting base in Wuzhi County, Henan Province on April 30, 2018, using local conventional planting standards. The tender leaf (L1), fully expanded leaf (L3), old leaf (L5), top of stem (S1), middle piece of stem (S2), lower stem (S3), seed stock (SS), head of tuberous root (HTR), and middle of tuberous root (MTR) of W85-5 plants were collected from six plants grown for about 6months. The floral organs including young flower bud (YB), mature flower bud (MaB), and fully opened flower (MF) were collected on 20 April of the following year. The samples used for RNA-Seq and qRT-PCR analysis were stored at −80°C, and the quality characteristics analysis materials were dried and crushed and stored in a desiccator.

### Identification of Putative WRKY mRNAs

The *R. glutinosa* transcript database obtained by RNA-Seq collected over 87,665 unigenes from *R. glutinosa* leaves and tuberous roots. The coding sequence (CDS) of WRKY genes from *A. thaliana* was used to determine homology in the *R. glutinosa* transcript database by basic local alignment (BLASTn). To remove redundancy, sequences were assembled using the SeqMan function of the DNAStar software package and manually adjusted. Only sequences that shared >95% of the matches are considered redundant. The open reading frames (ORFs) were predicted using ORF Finder^1^ and translated into amino acid sequences. Finally, to confirm that the obtained sequences were WRKY members, all primary identified non-redundant amino acid sequences of the WRKY members were submitted to the website http://pfam.sanger.ac.uk to prediction of the WRKY structural domain. Only the sequences that shared the WRKY domains were confirmed to be WRKY members. The *R. glutinosa* WRKY sequences reported in this work have been submitted to GenBank under accession numbers MZ285925-MZ285961.

### Protein Structure and Phylogenetic Analysis

To identify potential protein motifs in *R. glutinosa* WRKY, we used the MEME version 4.9.1 tool (http://meme-suite.org/tools/meme; Bailey et al., 2009) with the following parameters: The distribution of motifs was 0 or 1 per sequence; the maximum number of motifs was 10 motifs, the minimum motif width was 6, and the maximum width was 50. In addition, only motifs with e-value≤1e^−10^ were retained for further analysis. Subsequently, the detected motifs were searched in protein databases using the SMART (http://smart.embl.de/; Letunic et al., 2015) program. The amino acid sequences of *Arabidopsis* and rice WRKY proteins were downloaded from the NCBI database, and phylogenetic trees were constructed using the neighbor-joining method and bootstrap analysis (1,000 replicates) of MEGA 6.06 (www.megasoftware.net; Tamura et al., 2013).

### Expression Profile Analysis of WRKY Genes

The total RNA was extracted from hairy roots with elicitor treatment and 12 different tissues of *R. glutinosa* using TRIzol reagent (Invitrogen) according to the instruction and then treated with RNase-free DNaseI (Invitrogen). The RNA quality and concentration were determined using a Nanodrop™ 2000 spectrophotometer (Thermo Fisher Scientific, United States) and a Bioanalyzer 2100 (Agilent, United States). RNA-seq analysis of SA- and MeJA-treated hairy roots of *R. glutinosa* were conducted using Illumina HiSeq™ 2000 platform (Project ID: F15FTSCCKF2309). The H_2_O_2_-treated hairy roots (Project ID: F18FTSCCKF1733) and 12 *R. glutinosa* tissues (Project ID: F19FTSCCKF2067) were subjected to RNA-Seq analysis using BGISEQ-500 from BGI-Tech (Shenzhen, China). The RNA-Seq data (SRR5438036, SRR5438037, and SRR5438042) from SA-treated *R. glutinosa* hairy roots have been deposited in NCBI under project number PRJNA382479. RNA-seq data (CRA004677 CRA004688 and CRA004689) from MeJA- and H_2_O_2_-treated *R. glutinosa* hairy roots and 12 *R. glutinosa* tissues have been deposited in Genome Sequence Archive^2^ under project number PRJCA006052, PRJCA006054, and PRJCA006055.

Transcripts from tuberous roots and leaves transcriptomes of *R. glutinosa* (Project ID: F13FTSCCKF1467) were used as references for read mapping and gene annotation (Wang et al., 2017a). The clean reads from each sample were individually aligned to the reference transcripts of *R. glutinosa* using the Bowtie2 software (Langmead et al., 2009), and the abundance of gene transcripts was estimated using the RSEM method (Li and Dewey, 2011) and measured as fragments per kilobase of transcript per million fragments sequenced (FPKM; Trapnell et al., 2010). Genes with the FPKM fold change absolute value≥2 and controlling for false discovery rate adjusted *value* of *p*<0.001 were designated as differentially expressed genes. The expression profiles of DGEs from different samples were analyzed by hierarchical clustering, and a heat map of expression values was generated using the T-MeV 4.9.0 software (Howe et al., 2011).

### Quantitative Real-Time PCR Assays

Total RNA was extracted using TRIzol reagent (Invitrogen) according to the manufacturer’s instructions and treated with RNase-free DNase I (Invitrogen). cDNA was synthesized from 1μg of total RNA using the PrimeScript™ II 1st Strand cDNA Synthesis Kit (TaKaRa Bio, Dalian). Relative expression levels of genes were analyzed by qRT-PCR using SYBR® Premix Ex Taq™ II (Tli RNaseH Plus; Takara Bio, Dalian) on a Bio-Rad iQ5 Real-Time PCR System (Bio-Rad, United States), as described by Wang et al. (2017a). The *RgTIP41* gene (GenBank accession number KT306007) was used as an endogenous control to normalization relative expression levels based on the 2^-ΔΔCt^ method (Schmittgen and Livak, 2008). Data were analyzed using ANOVA and Student’s *t* test (*p*<0.05). The specific primer sequences used in this study are listed in Supplementary Table S1.

### Determination of Acteoside and Total Phenylethanoid Glycosides

The content of acteoside in the hairy roots and different tissues of *R. glutinosa* was determined according to a high-performance liquid chromatographic (HPLC) method established by the group (Wang et al., 2017a). Total PhG content in the samples was determined using a UV spectrophotometer, referring to the method established in a previous study (Yi et al., 2017).

### Vector Construction and Transformation

For overexpression of *RgWRKY37* vectors, primers of the complete *RgWRKY37* ORF sequences were designed using Primer-BLAST online, software,^3^ and the sequences are shown in Supplementary Table S1. The *RgWRKY37* sequence was amplified using PrimeSTAR® HS DNA Polymerase (Takara, Dalian), and the amplification product was purified. The purified product was subsequently cloned into a pBI121 to construct the *RgWRKY37* overexpression vector, which is driven by the CaMV35S promoter with a fragment of the neomycin phosphotransferase II (NPTII) gene, conferring resistance to kanamycin. The recombinant vector was transformed into the *Agrobacterium rhizogenes* strain MSU440 using the freeze-thaw method. Specific primers (Supplementary Table S1) were designed to amplify the complete *RgWRKY37* ORF sequence without the stop codon. The amplified fragment was inserted into the pBWA(V)HS-GLosgfp vector with a CaMV35S promoter and fused to the N-terminal region of the green fluorescent protein (GFP) gene, to generate the CaMV35S:*RgWRKY37*-GFP recombinant plasmid, and transformed it into the *Agrobacterium tumefaciens* LBA4404 strain using the freeze-thaw method.

### Generation of Transgenic *R. glutinosa* Hairy Roots

The *A. rhizogenes* MSU440 with *RgWRKY37*-OE vector was initiated from glycerol stock and grown overnight in LB liquid medium at 28°C with shaking (180rpm) until OD_600_ was 0.6. Leaves from 25-day-old seedings of *R. glutinosa* were cut into 0.5×0.5 leaf disks and infected with *A. rhizogenes* MSU440 liquid inoculation medium for 5min. The infested leaf disks were placed on MS solid medium containing 100μmol/l acetosyringone (AS) and incubated in the dark at 26°C for 2days. After coculture, the leaf explants were transferred to MS solid medium containing 250mg/L timentin and 100μmol/L AS for hairy roots induction. Numerous hairy roots were observed emerging from the wound sites after approximately 2–4weeks. When the hairy roots had grown to a length of 2–3cm, they were separated from the explants and cultured in the dark at 26°C on MS medium with a gradual decrease in timentin at a 20-day interval to obtain bacteria-free culture. Well-grown hairy root was inoculated in flasks containing 50ml MS liquid medium and cultured on a gyratory shaker at 120rpm in the dark (26°C). Total DNA was isolated from *R. glutinosa* hairy roots and non-transformed (NT) hairy roots cultured in suspension for 50days using a modified CTAB method (Cuc et al., 2008). The DNA was used as template for transgenic identification by PCR with specific primers for pBI121-D and rolB-D (Supplementary Table S1). The amplification system and procedure were identical to that of Wang et al. (2017a).

### Correlation Analysis of Genes and Metabolites

To construct gene-metabolite regulatory network of *RgWRKY37*, the *RgWRKY37* overexpressed hairy root and wild-type (WT) hairy root cultured for 40days were used to RNA-seq analysis, respectively. The RNA-Seq data (CRA004786) from *RgWRKY37* overexpressed *R. glutinosa* hairy roots have been deposited in Genome Sequence Archive under project number PRJCA006249. Correlations between the WRKY genes, enzyme genes, and acteoside were calculated using the Pearson correlation coefficient using DPS v7.05 software (Tang and Zhang, 2013) based on the co-occurrence principle between mRNA and metabolite levels. The acteoside content and the FPKM values of *RgWRKY37* and key enzyme genes in acteoside biosynthesis were used to construct coexpression networks. The expression correlation matrix was generated with Cytoscape software (Shannon et al., 2003) to measure the similarity of expression between pairwise genes. Gene pairs with *r*>0.60 (positive coexpression) or *r*<−0.60 (negative coexpression) were considered significantly coexpressed.

### Determination of Subcellular Localization

Protoplasts were prepared from *Nicotiana benthamian* mesophyll cell and transformed, according to previously described method (Yoo et al., 2007). The protoplasts were transformed with highly purified *RgWRKY37*-GFP-containing plasmid DNA. After 24h of transformation, the protoplasts were observed under a laser confocal scanning microscope (Olympus FV10-ASW, Tokyo, Japan), and the protoplasts expressing GFP alone were used as control.

## Results

### Identification of the WRKY Gene Family in *R. Glutinosa*

In order to predict the WRKY gene of *R. glutinosa*, 71 WRKY gene cDNA sequences of *Arabidopsis* were downloaded from GenBank^4^ as search sequences. A total of 115 unigenes were retrieved in the *R. glutinosa* transcriptome database by BLASTn. There were 47 sequences with bases between 200 and 500bp and 68 sequences larger than 500bp. Online prediction of the 68 sequences larger than 500bp was performed using the online tool ORF Finder.^5^ The results showed that 44 cDNA sequences had complete ORFs, and 37 WRKY gene cDNA sequences were eventually obtained after removing four redundant sequences (Table 1). It can be seen that the length of the 37 identified *WRKY* genes of *R. glutinosa* ranges from 835 to 2,541bp, and the predicted amino acid sequence length ranges from 192 to 712 aa. Analysis using Batch CD (CDD) search software and SMART software confirmed that all 37 sequences with complete ORFs were all cDNA of the *WRKY* gene.

**Table 1:** WRKY genes identified in *R. glutinosa* transcriptome.

Gene name	Gene ID	Gene length/bp	Amino acid length	Blast results [Query cover, E-value, Identities, Accession No., and Description (species)]
*RgWRKY1*	CL672.Contig4	2,541	712	100%, 0.0, 74%, XP_011095658.1, and probable WRKY transcription factor 2 (*Sesamum indicum*)
*RgWRKY2*	CL672.Contig2	2,445	696	100%, 0.0, 73%, XP_011097672.1, and probable WRKY transcription factor 2 isoform X1 (*S. indicum*)
*RgWRKY3*	CL2462.Contig2	2,169	489	100%, 0.0, 76%, XP_011098678.1, and probable WRKY transcription factor 4 (*S. indicum*)
*RgWRKY4*	CL7634.Contig3	1748	484	96%, 0.0, 69%, XP_011070970.1, and probable WRKY transcription factor 3 isoform X2 (*S. indicum*)
*RgWRKY5*	CL2462.Contig4	1911	489	99%, 0.0, 70%, XP_011085706.1, and probable WRKY transcription factor 4 isoform X1 (*S. indicum*)
*RgWRKY6*	CL7634.Contig2	1903	508	96%, 0.0, 76%, XP_011070970.1, and probable WRKY transcription factor 3 isoform X2 (*S. indicum*)
*RgWRKY7*	CL6341.Contig1	1793	498	100%, 0.0, 66%, XP_012832231.1, and probable WRKY transcription factor 26 (*Erythranthe guttatus*)
*RgWRKY8*	Unigene18958	1829	570	100%, 0.0, 85%, XP_011095503.1, and probable WRKY transcription factor 20 (*S. indicum*)
*RgWRKY9*	CL6521.Contig2	1912	440	94%, 0.0, 71%, XP_011080757.1, and probable WRKY transcription factor 26 (*S. indicum*)
*RgWRKY10*	CL3420.Contig1	1,677	429	98%, 0.0, 66%, XP_011094753.1, and WRKY transcription factor 44-like (*S. indicum*)
*RgWRKY11*	Unigene13860	2,531	653	94%, 0.0, 69%, XP_011100622.1, and WRKY transcription factor 1-like isoform X1 (*S. indicum*)
*RgWRKY12*	CL1859.Contig2	1830	499	93%, 0.0, 69%, XP_011083976.1, and probable WRKY transcription factor 32 (*S. indicum*)
*RgWRKY13*	Unigene20949	1,331	313	100%, 3e-136, 71%, XP_011085274.1, and probable WRKY transcription factor 71 isoform X1 (*S. indicum*)
*RgWRKY14*	Unigene18730	1,318	312	91%, 1e-88, 60%, XP_011079532.1, and probable WRKY transcription factor 23 (*S. indicum*)
*RgWRKY15*	Unigene21765	1,167	292	100%, 8e-98, 59%, XP_011085274.1, and probable WRKY transcription factor 71 isoform X1 (*S. indicum*)
*RgWRKY16*	Unigene9802	982	241	100%, 5e-104, 72%, XP_011101863.1, and probable WRKY transcription factor 12 (*S. indicum*)
*RgWRKY17*	Unigene1594	835	243	98%, 2e-122, 78%, AKA27885.1, and WRKY protein (*Salvia miltiorrhiza*)
*RgWRKY18*	CL9158.Contig2	1,437	364	80%, 4e-90, 63%, XP_011100744.1, and probable WRKY transcription factor 48 (*S. indicum*)
*RgWRKY19*	CL1659.Contig1	1,486	334	100%, 6e-136, 74%, XP_011070956.1, and probable WRKY transcription factor 57 isoform X2 (*Sesamum indicum*)
*RgWRKY20*	CL6621.Contig3	1,454	335	100%, 0.0, 79%, XP_011080000.1, and probable WRKY transcription factor 21 isoform X1 (*S. indicum*)
*RgWRKY21*	CL6621.Contig2	1,445	332	100%, 0.0,77%, XP_011080000.1, and probable WRKY transcription factor 21 isoform X1 (*S. indicum*)
*RgWRKY22*	CL4390.Contig1	1,164	312	99%, 3e-158, 71%, XP_011077400.1, and probable WRKY transcription factor 11 (*S. indicum*)
*RgWRKY23*	Unigene12117	998	192	98%, 5e-75, 63%, XP_009343134.1, and probable WRKY transcription factor 75 (*Pyrus bretschneideri*)
*RgWRKY24*	CL4612.Contig2	1,518	356	81%, 2e-132, 74%, XP_011099177.1, and probable WRKY transcription factor 7 (*S. indicum*)
*RgWRKY25*	CL9362.Contig2	1,648	343	100%, 0.0, 82%, XP_011082056.1, and probable WRKY transcription factor 17 (*S. indicum*)
*RgWRKY26*	CL8843.Contig2	1,456	318	100%, 0.0, 87%, XP_011074403.1, and probable WRKY transcription factor 7 (*S. indicum*)
*RgWRKY27*	CL5269.Contig1	854	160	96%, 1e-52, 59%, XP_020554654.1, and probable WRKY transcription factor 51 (*S. indicum*)
*RgWRKY28*	Unigene2387	1,472	348	99%, 2e-170, 72%, XP_011073725.1, and probable WRKY transcription factor 53 (*S. indicum*)
*RgWRKY29*	CL4123.Contig4	1709	343	100%, 3e-170, 68%, XP_011101023.1, and probable WRKY transcription factor 53 (*S. indicum*)
*RgWRKY30*	CL805.Contig2	2095	549	100%, 0.0, 74%, XP_011096037.1, and WRKY transcription factor 6 (*S. indicum*)
*RgWRKY31*	Unigene7844	1,322	329	99%, 1e-140, 67%, XP_011081142.1, and probable WRKY transcription factor 29 (*S. indicum*)
*RgWRKY32*	Unigene16209	1,126	210	99%, 8e-55, 50%, PIN12289.1, and hypothetical protein, (*Handroanthus impetiginosus*)
*RgWRKY33*	CL4160.Contig2	1,355	365	100%, 3e-137, 61%, XP_011076029.1, and probable WRKY transcription factor 30 (*S. indicum*)
*RgWRKY34*	Unigene18239	1,053	262	97%, 2e-121, 76%, XP_011073326.1, and probable WRKY transcription factor 40 (*S. indicum*)
*RgWRKY35*	CL3622.Contig2	1,434	297	95%, 4e-138, 69%, XP_011081961.1, and probable WRKY transcription factor 40 (*S. indicum*)
*RgWRKY36*	Unigene20977	1,058	292	99%, 1e-127, 74%, XP_011101421.1, and probable WRKY transcription factor 65 (*S. indicum*)
*RgWRKY37*	CL394.Contig2	1,303	312	97%, 5e-97, 52%, XP_012832290.1, and probable WRKY transcription factor 70 (*E. guttatus*)

The predicted amino acid sequence of WRKY in *R. glutinosa* showed a homologous alignment on NCBI, and it was found that 36 sequences had the highest consistency with the WRKY sequence of *Sesamum indicum*, a plant of the family Pedaliaceae. RgWRKY17 has the highest sequence identity with the homologous protein of *S. miltiorrhiza* homologous protein from Labiatae. RgWRKY7 and RgWRKY37 have the highest sequence identity with the homologous protein of *Erythranthe guttata*, which also belongs to Scrophulariaceae. Pedaliaceae, Labiatae, and Scrophulariaceae all belong to the Sympetalae subclass of Tubiflorae. RgWRKY23 and RgWRKY32 have the highest sequence identity with *Pyrus bretschneideri* from Rosaceae and *Handroanthus impetiginosus* from Bignoniaceae, respectively.

### Phylogenetic Analysis of the WRKY Proteins

**Figure 1:**
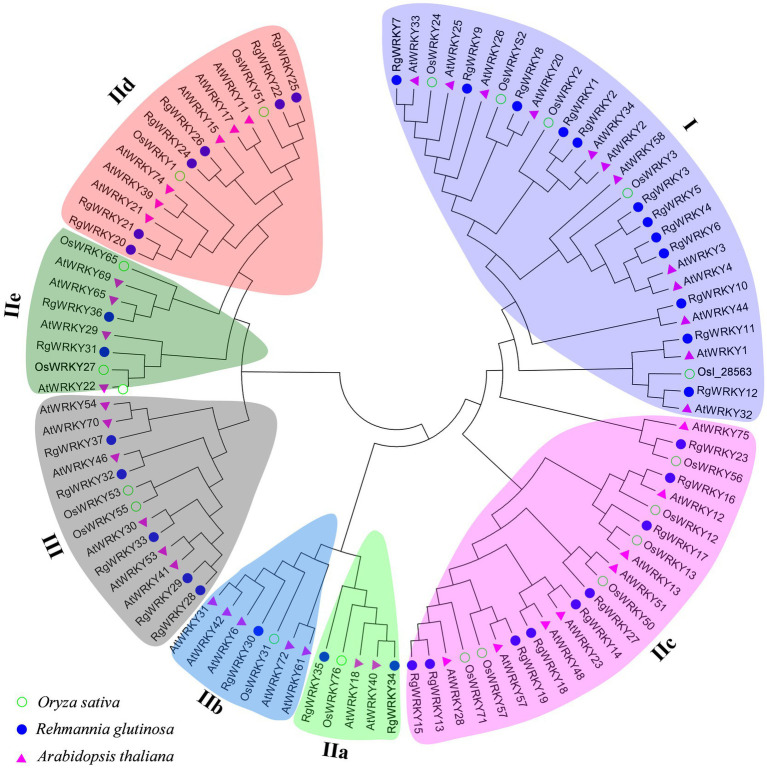
Phylogenetic tree of the WRKY transcription factors from *Rehmannia glutinosa*, *Arabidopsis* and rice. The full-length sequences of the WRKY proteins were aligned using MAFFT, and the phylogenetic tree was constructed using the neighbor-joining method in the MEGA6 software (Tamura et al., 2013). Bootstrap values (shown at the corresponding nodes) were obtained from 1,000 replicates and are reported as percentages. The black arcs indicate different groups (or subgroups) of WRKY domains.

To further examine the evolutionary relationships among *R. glutinosa*, *Arabidopsis*, and rice WRKY proteins, multiple sequence alignments of the complete amino acid sequences of all WRKY proteins were performed using the MAFFT program. An unrooted phylogenetic tree (Figure 1) was then constructed using the neighbor-joining method of MEGA6.06 (Tamura et al., 2013) with the multiple sequence alignment files. Based on the number of WRKY domains (WDs) and the features of the specific zinc finger motifs, all 37 *RgWRKY* genes were classified into three main groups, with five subgroups in group II. Twelve *R. glutinosa* WRKY genes (*RgWRKY1* to *RgWRKY12*) with two WRKY domains belong to group I and have a zinc finger motif of C–X_4_–C–X_22-23_–H–X_1_–H. The other 20 *R. glutinosa* WRKY proteins with the zinc finger structure of C–X_4-5_–C–X_23_–H–X_1_–H were assigned to group II, which comprised 54% of the total number of *RgWRKY* genes. The 20 *RgWRKY* genes of group II are unevenly distributed among the five subgroups: group IIa (two: *RgWRKY34*, *RgWRKY35*), group IIb (one: RgWRKY30), group IIc (nine: *RgWRKY13*, *RgWRKY14*, *RgWRKY15*, RgWRKY16, *RgWRKY17*, *RgWRKY18*, *RgWRKY19*, *RgWRKY23*, and *RgWRKY27*), group IId (six: *RgWRKY20*, *RgWRKY21*, *RgWRKY22*, *RgWRKY24*, *RgWRKY25*, and *RgWRKY26*), and group IIe (two: *RgWRKY31*, *RgWRKY36*). In contrast to group I, group II genes have only one WRKY domain. Instead of the C_2_H_2_ pattern, group III genes contain a C_2_HC zinc finger motif (C–X_7_–C–X_23_–H–X_1_–C) and five of the 37 *RgWRKY* genes (*RgWRKY28*, *RgWRKY29*, *RgWRKY32*, *RgWRKY33*, and *RgWRKY37*) belong to this group. Detailed information about the classification of the genes and the WRKY domains and the profile of zinc finger motifs can be found in Table 1.

### Protein Motifs and Structure Analysis of the WRKYs in *R. glutinosa*

**Figure 2:**
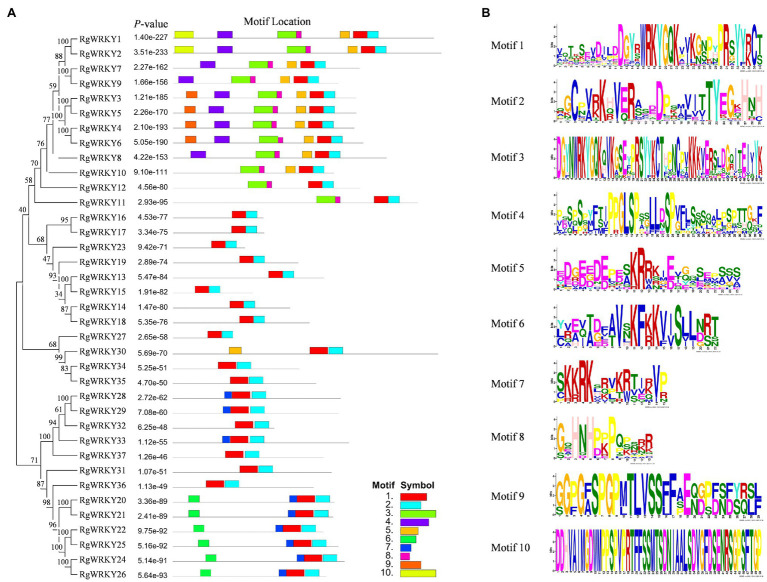
Phylogenetic clustering and conserved protein motifs of *RgWRKY* genes. **(A)** Phylogenetic relationships and motif compositions of RgWRKY members. The phylogenetic tree was constructed with the full-length sequences of *R. glutinosa* WRKY proteins using MEGA v6.0. **(B)** Ten types of conserved motifs in *R. glutinosa*. The conserved motifs were predicted using the MEME program.

To better understand the conservation and diversification of the *R. glutinosa* WRKY proteins, the putative motifs of all WRKY proteins were predicted by MEME motif analysis, and 10 distinct motifs were identified. The locations of the WRKY domains, zinc finger binding motifs, and any conserved motifs are shown in Figure 2. Motif 1 plus Motif 2 was found in all 37 WRKY proteins, and Motif 3 plus Motif 8 was found only in the N-terminal of 12 WRKY proteins. SMART analysis revealed that Motif 1 plus Motif 2 and Motif 3 plus Motif 8 are conserved WRKY domains and zinc finger motifs.

**Figure 3:**
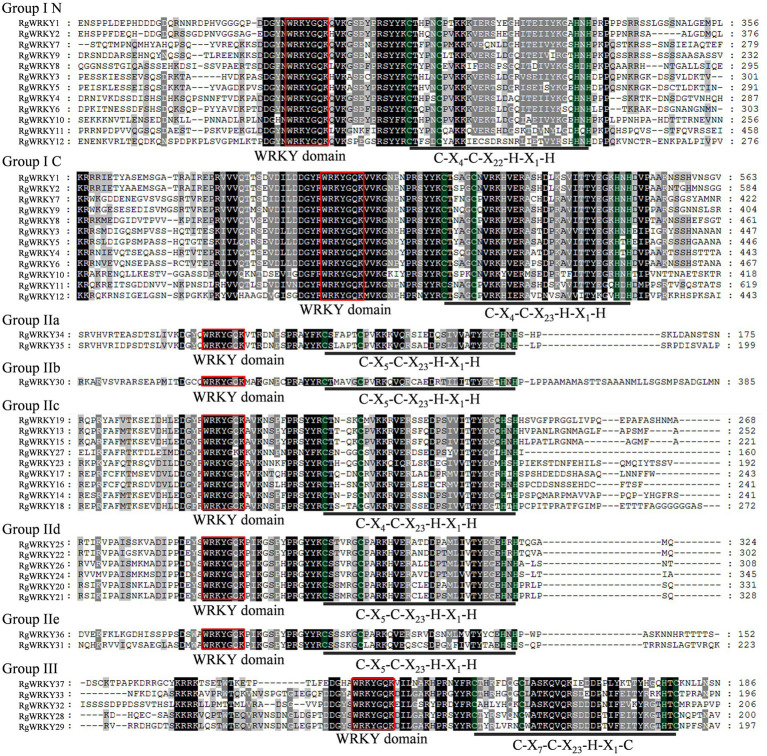
Multiple sequence alignment of the WRKY domain among *R. glutinosa* WRKY genes. Red indicates conserved WRKY amino acid domains; green indicates zinc finger motifs; and dashes indicate gaps. “N” and “C” indicate the N-terminal and C-terminal WRKY domains of a specific WRKY gene.

The most prominent structural feature of WRKY proteins is the WRKY domain, which has been shown to interact with the W-box (C/T)TGAC(T/C) to activate a large number of defense-related genes (Eulgem et al., 2000). The WRKY domain consists of a highly conserved WRKYGQK heptapeptide stretch at the N-terminus, followed by a zinc finger motif (Eulgem et al., 2000). A multiple sequence alignment of the core WRKY domain, spanning approximately 60 amino acids of all 37 RgWRKY proteins, is shown in Figure 3. A total of 36 RgWRKY proteins were found to have highly conserved WRKYGQK sequences, while RgWRKY27 varies by a single amino acid and has the variant WRKYGKK sequence, which is consistent with two *Lilium regale* Group IIc members (LrWRKY6 and LrWRKY7; Cui et al., 2018). It is hypothesized that variation in this WRKY domain may alter the binding specificity in the DNA targets, but this remains to be demonstrated. As previously described (Eulgem et al., 2000), the metal-chelating zinc finger motif (C–X_4-5_–X_22-23_–H–X_1_–H or C–X_7_–C–X_23_–H–X_1_–C) is another important structural characteristic of WRKY proteins. Interestingly, all zinc finger motifs of WRKR group II are C–X_7_–C–X_23_–H–X_1_–C: In contrast to group III WRKY in rice (Wu et al., 2005) and barley (Mangelsen et al., 2008), there are no RgWRKY in group III proteins containing a C–X_7_–C–X_24_–H–X_1_–C zinc finger motif, which have the same domain characteristic as the grape WRKR group III proteins, perhaps suggesting that this may be a feature of monocotyledonous species.

### Expression Profiles of *R. glutinosa* WRKY Genes Under SA and MeJA Treatments

**Figure 4:**
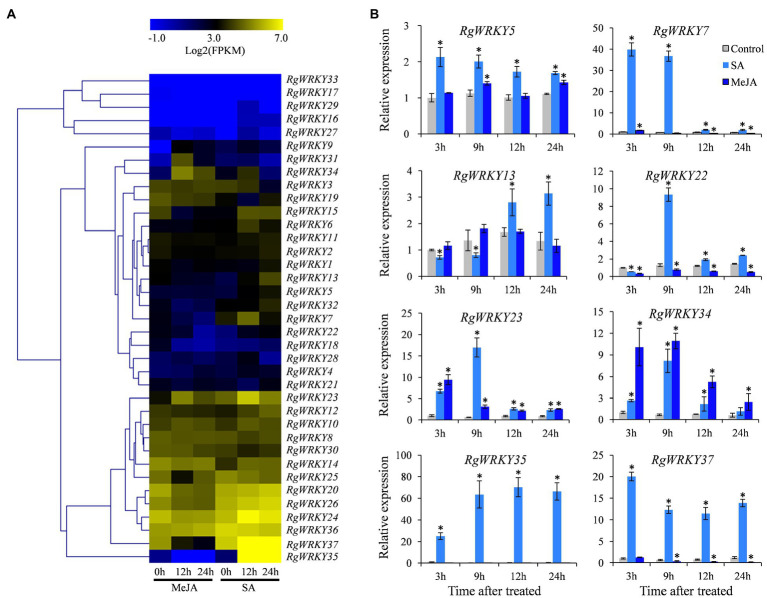
Expression profiles of 37 WRKY genes in *R. glutinosa* hairy roots under salicylic acid (SA) and methyl jasmonate (MeJA) treatments. **(A)** Heat map representing expression dynamics of WRKY genes after SA and MeJA treatments. The expression value (FPKM) for unigenes was log2-transformed and scaled across each row, and heat map was generated by MultiExperiment Viewer (MeV). **(B)** Relative expression levels of eight WRKY genes in hairy roots after SA and MeJA treatments. These genes expression levels were all determined by quantitative real-time PCR (qRT-PCR). Vertical bars indicate the SD of three biological replicates. Asterisks indicate a significant difference at the *p*<0.05 level. The same as follows.

Salicylic acid and MeJA play important regulatory roles as signaling molecules in plant response to adversity stress and the accumulation of secondary metabolites (Pieterse et al., 2009; Danaee et al., 2015). Our previous research found that SA and MeJA could promote the accumulation of acteoside in the *R. glutinosa* hairy roots at appropriate concentrations (Wang et al., 2017a). Relative to untreated hairy roots, SA induced significant increase in acteoside content at a wide range of concentration (10 – 40μmol/L), but MeJA only slightly increased accumulation of acteoside at lower concentration (5μmol/L). To identify WRKY genes in response to SA and MeJA treatments, the expression levels of these 37 *RgWRKY* genes were determined by comparing 12 and 24h samples with the control sample suing RNA-seq analysis. The results indicate that these *RgWRKY* genes showed differential expression patterns under SA and MeJA treatments in hairy roots of *R. glutinosa* (Figure 4A; Supplementary Table S2). Twelve and 10 *RgWRKY* genes were found to be differentially expressed at atleast one time point after MeJA and SA treatments, respectively. Four genes, *RgWRKY13*, *RgWRKY15*, *RgWRKY35*, and *RgWRKY37*, were differentially expressed and upregulated at 12 and 24h after SA treatment, and the log2 (0/12h) and log2 (0/24h) for *RgWRKY35* and *RgWRKY37* were both greater than 2.3. There were six genes differentially expressed at 12 and 24h after MeJA treatment. Among them, *RgWRKY9*, *RgWRKY31*, and *RgWRKY34* were all upregulated, and their log2 (0/12h) and log2 (0/24h) were greater than 2.0, while *RgWRKY15*, *RgWRKY18*, and *RgWRKY37* were all downregulated. Interestingly, both *RgWRKY15* and *RgWRKY37* were differentially expressed after SA and MeJA treatments, showing upregulated expression after SA treatment but inhibited expression after MeJA treatment. In addition, *RgWRKY34* significantly upregulated differential expression at 12h after SA treatment and two time points after MeJA treatment, and *RgWRKY23* was significantly differentially expressed at 12h after SA and MeJA treatment.

Based on their expression patterns of the 37 *RgWRKY* genes in *R. glutinosa* hairy roots under SA and MeJA treatments, eight *RgWRKY* genes were selected for qRT-PCR analysis to detect their relative expression levels at 3, 9, 12, and 24h after SA and MeJA treatment. Similar to the RNA-seq analysis, qRT-PCR results showed that eight *RgWRKY* genes were significantly upregulated at 12 and 24h after SA treatment (Figure 4B). Among them, *RgWRKY5*, *RgWRKY7*, *RgWRKY22*, *RgWRKY23*, and *RgWRKY34* genes had the highest expression levels at 3 or 9h after SA treatment, and *RgWRKY13* had higher expression levels at 12 and 24h after SA treatment. The expression levels of *RgWRKY35* and *RgWRKY37* genes showed a highly significant increase after SA treatment, while the expression levels of *RgWRKY37* increased by 10.45-fold–19.03-fold, and *RgWRKY35* increased by 23.92-fold–69.27-fold. After MeJA treatment, the expression levels of *RgWRKY23* and *RgWRKY34* were significantly upregulated at 4h, and the other six *RgWRKY* genes were all insensitive to the induction or downregulated. *RgWRKY23* and *RgWRKY34* are two genes that simultaneously respond to SA and MeJA treatments. In addition, the expression of *RgWRKY27* gene was detected by qRT-PCR, the log2 (0/12h) and log2 (0/24h) of which were greater than 6.0, but the statistical value was not significant after SA treatment. The results of qRT-PCR analysis showed that the expression level of *RgWRKY27* in *R. glutinosa* hairy roots was significantly higher than that of the control at 3, 12, and 24h after SA treatment (Supplementary Figure S2). The results of the above expression patterns suggest that different *WRKY* genes may participate in the biosynthesis of acteoside by responding to different elicitors.

### Expression Profiles of *R. glutinosa* WRKY Genes Under H_2_O_2_ Treatment

**Figure 5:**
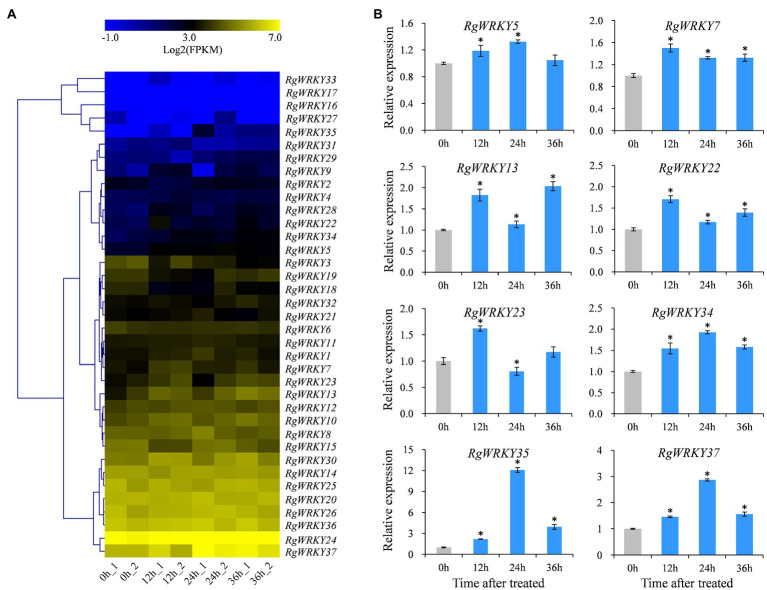
Expression profiles of 37 WRKY genes in *R. glutinosa* hairy roots after H_2_O_2_ treatment. **(A)** Heat map representing expression dynamics of WRKY genes after H_2_O_2_ treated. **(B)** Relative expression levels of eight WRKY genes in hairy roots after H_2_O_2_ treatment by qRT-PCR. The asterisks indicate statistically significant differences at *p*<0.05.

Hydrogen peroxide (H_2_O_2_), as a phytohormone, plays important roles in regulating plant growth and development (Černý et al., 2018). In previous studies, exogenous application of H_2_O_2_ induced SA biosynthesis in *Nicotiana tabacum* leaves (Leon et al., 1995). In addition, SA treatment promotes the production of H_2_O_2_ in *A. thaliana* (Rao et al., 1997). The content of acteoside in the hairy roots of *R. glutinosa* was detected at 12, 24, and 36h after H_2_O_2_ treatment, and it was found that the acteoside content at 24 and 36h after H_2_O_2_ treatment was 1.52 and 1.84 times that of the control, respectively (Supplementary Figure S3). RNA-seq analysis showed that 115, 77, and 123 DGEs in Control-*vs*-H12, Control-*vs*-H24, and Control-*vs*-H36 were enriched in the KEGG pathway of phenylpropanoid biosynthesis, respectively (Supplementary Figure S4, Supplementary Table S3). Overall, 37 *WRKY* genes had different response patterns to H_2_O_2_ induction (Figure 5A, Supplementary Table S4). There were four, three, and six *WRKY* genes that were differentially expressed at 12, 24, and 36h after H_2_O_2_ treatment, respectively. Only the *RgWRKY33* gene was differentially expressed at the three time points after H_2_O_2_ treatment. *RgWRKY9* and *RgWRKY35* were significantly upregulated at least two time points after H_2_O_2_ treatment. Furthermore, it was found that *RgWRKY13*, *RgWRKY22*, *RgWRKY28*, *RgWRKY34*, and *RgWRKY37* were significantly upregulated at least two time points after H_2_O_2_ treatment. qRT-PCR analysis revealed that *RgWRKY34*, *RgWRKY35*, and *RgWRKY37* showed similar expression profiles under H_2_O_2_ treatment and had the highest expression levels at 24h after H_2_O_2_ treatment (Figure 5B).

### Expression Patterns of *R. glutinosa* WRKY Genes in Various Tissues of *R. glutinosa* Plants

**Figure 6:**
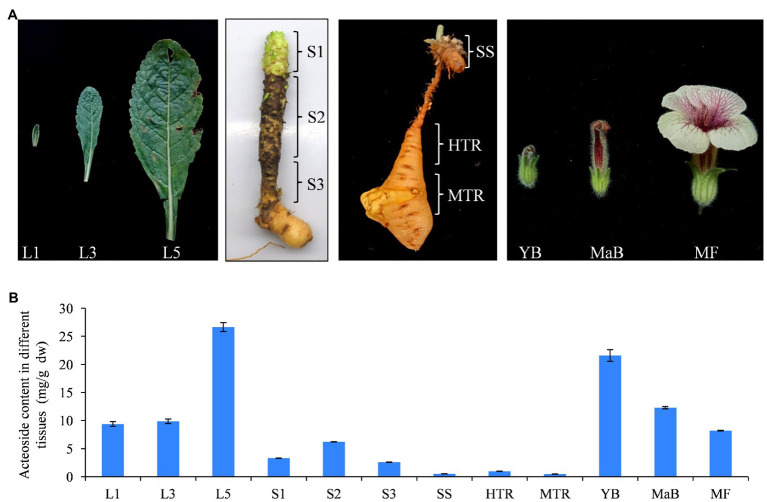
Acteoside content in different tissues of *R. glutinosa*. **(A)** Characteristics of the 12 tissues of *R. glutinosa*. **(B)** Acteoside content in 12 tissues of *R. glutinosa* plants. L1, tender leaf; L3, fully expanded leaf; L5, old leaf; S1, top of stem; S2, middle piece of stem; S3, lower stem; SS, seed stock; HTR, the head of tuberous root; MTR, the middle of tuberous root; YB, Young flower bud; MaB; mature flower bud; and MF, newly opened flower.

In previous studies, we found that the content of medicinal ingredients (i.e., catalpol and acteoside) presented typical tissue characteristics (Wang et al., 2017b; Zhi et al., 2018). We used HPLC method to detect the acteoside content in different *R. glutinosa* tissues, including tender leaf (L1), fully expanded leaf (L3), old leaf (L5), top of stem (S1), middle piece of stem (S2), lower stem (S3), SS, HTR, MTR, YB, MaB, and MF. The determined results indicated that the acteoside contents were higher in leaves and floral organs than in stems, seed stock, and tuberous roots (Figure 6).

**Figure 7:**
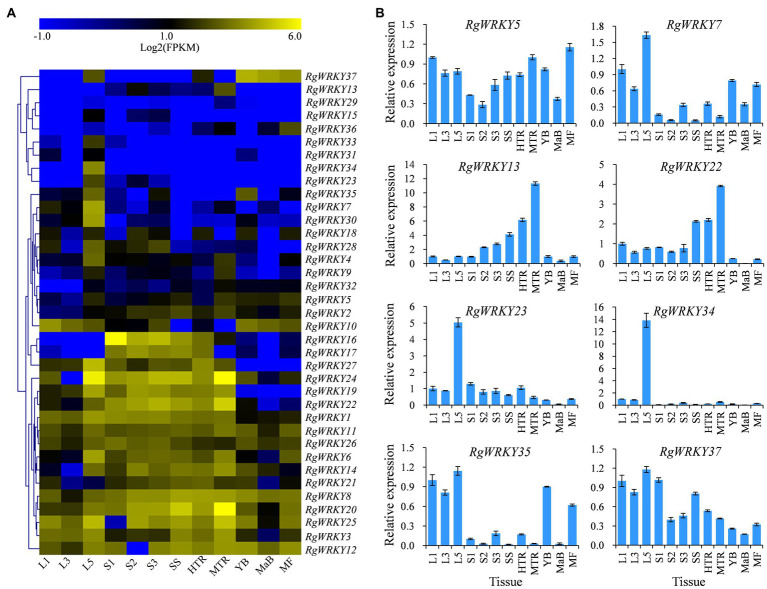
Expression patterns of 37 WRKY genes in various tissues of *R. glutinosa*. **(A)** Heat map representing expression dynamics of WRKY genes in different tissues. **(B)** Relative expression levels of WRKY genes in different tissues by qRT-PCR. L1, tender leaf; L3, fully expanded leaf; L5, old leaf; S1, top of stem; S2, middle piece of stem; S3, lower stem; SS, seed stock; HTR, the head of tuberous root; MTR, the middle of tuberous root; YB, Young flower bud; MaB; mature flower bud; and MF, fully opened flower.

To investigate the tissue-specific expression of *R. glutinosa* WRKY genes, RNA-seq analysis was used to determine the expression patterns of 37 *RgWRKY* genes in these tissues. The results showed that the expression of all 37 *RgWRKY* genes was detected in at least one of the 12 examined tissues with FPKM values greater than 1 (Supplementary Table S5). Among them, five genes, *RgWRKY1*, *RgWRKY8*, *RgWRKY11*, *RgWRKY20*, and *RgWRKY26*, showed high expression (|log2(FPKM)|>1) in all 12 tissues and organs (Figure 7, Supplementary Table S5). The expression pattern of *RgWRKY* genes indicated that many of them showed a relatively high expression in the old leaf (Figure 7A). Interestingly, the expression of key enzyme genes, including *RgC3H*, *RgC4H*, *RgCuAO*, *RgHCT*, *RgPAL*, *RgPPO*, *RgTyDC*, and *RgUGT* (CL592.Contig1), in the acteoside biosynthesis pathway, was higher in the old leaf (Supplementary Table S6). The results of qRT-PCR analysis showed that *RgWRKY23* and *RgWRKY34* were predominantly expressed in old leaf (Figure 7B). *RgWRKY7*, *RgWRKY35*, and *RgWRKY37* were more highly expressed in old leaf (Figure 7B), which is similar to the acteoside content in various tissues of *R. glutinosa*. Correlation analysis of acteoside content and gene expression levels of the 12 tissues of *R. glutinosa* revealed that the FPKM values of 10 *RgWRKY* genes were positively correlated to acteoside content (Supplementary Table S7). However, *RgWRKY13* and *RgWRKY22* showed high expression levels in seed stocks and tuberous roots, especially in the MTRs (Figure 7B), which was obviously different from the accumulation pattern of the acteoside. In conclusion, these results suggest that *RgWRKY7*, *RgWRKY23*, *RgWRKY34*, *RgWRKY35*, and *RgWRKY37* might be involved in the biosynthesis of acteoside.

### Overexpression of *RgWRKY37* in *R. glutinosa* Hairy Roots Increase Acteoside Content

**Figure 8:**
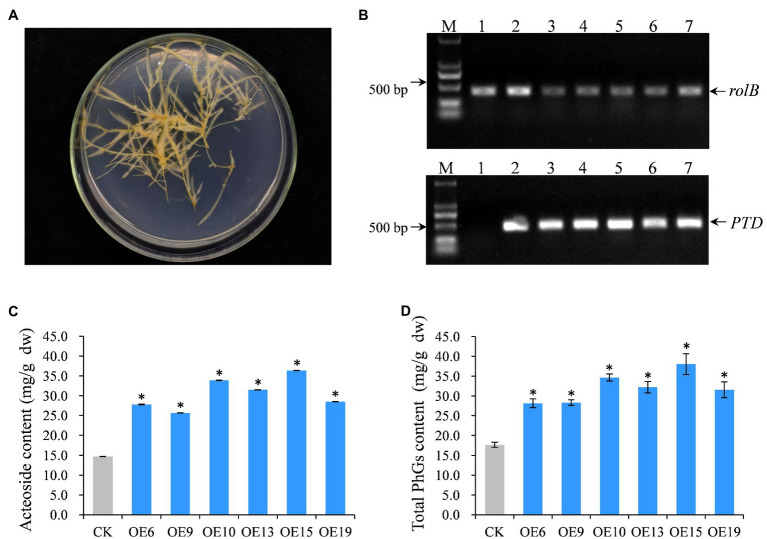
Functional investigation of the *RgWRKY37* in hairy root of *R. glutinosa*. **(A)** Phenotype of *RgWRKY37* overexpressed hairy roots. **(B)** PCR detection of transgenic *R. glutinosa* hairy roots using primers of *rolB* and pBI121. Lane 1, wild-type hairy roots; Lanes 2–7, *35S*-*RgWRKY37* hairy root lines; and M, DNA Marker. **(C)** Acteoside content in *35S*-*RgWRKY37* hairy roots. **(D)** Total phenylethanoid glycosides (PhGs) in *35S*-*RgWRKY37* hairy roots. The vertical bars show the SD values (*n*=3). The asterisks indicate statistically significant differences at *p*<0.05.

Based on the correlation analysis of expression levels of the 37 *RgWRKY* genes and acteoside content in hairy roots of *R. glutinosa* under SA and H_2_O_2_ treatments (Supplementary Tables S8, S9) and their correlation coefficients in 12 tissues of *R. glutinosa* plants (Supplementary Table S7), we found the expression pattern of *RgWRKY37* was highly associated with acteoside content, so we selected *RgWRKY37* for further studies to verify the functions of WRKY genes in acteoside biosynthesis. We constructed an overexpressing *RgWRKY37* recombinant plasmid driven by the CaMV 35S promoter and transformed it into hairy root *via Agrobacterium tumefaciens*-mediated transformation. In total, 19 transgenic hairy root lines of *R. glutinosa* were obtained. Among them, six transgenic *R. glutinosa* hairy root lines grew well in MS medium containing kanamycin (Figure 8A) and were confirmed as positive lines by PCR analysis (Figure 8B). It was observed that the hairy root lines overexpressing the *RgWRKY37* gene were slight darker than wild-type hairy root lines (Supplementary Figure S5A), and it suggest that the overexpressed *RgWRKY37* influenced the development and secondary metabolism of *R. glutinosa* hairy roots. We also found that the dry weight of the *35S*-*RgWRKY37* hairy root lines was varied (Supplementary Figure S5B), and it was probably that growth rate of the different hairy root lines was inconsistent with each other; this phenotype may be irrelevant to *RgWRKY37*. To further analysis the function of *RgWRKY37* in acteoside biosynthesis, the content of acteoside in transgenic and wild-type hairy root lines was determined by HPLC. The result showed that the acteoside content of the six tested transgenic hairy roots increased by 74.44–147.11% compared to the wild type (Figure 8C). Furthermore, the total PhGs content was significantly increased in the *R. glutinosa* hairy roots of *RgWRKY37* overexpressed (Figure 8D). The results showed that overexpression of *RgWRKY37* could enhance the accumulation of acteoside and total PhGs in *R. glutinosa* hairy roots.

### Regulator Network of *RgWRKY37* on Acteoside Biosynthesis

**Figure 9:**
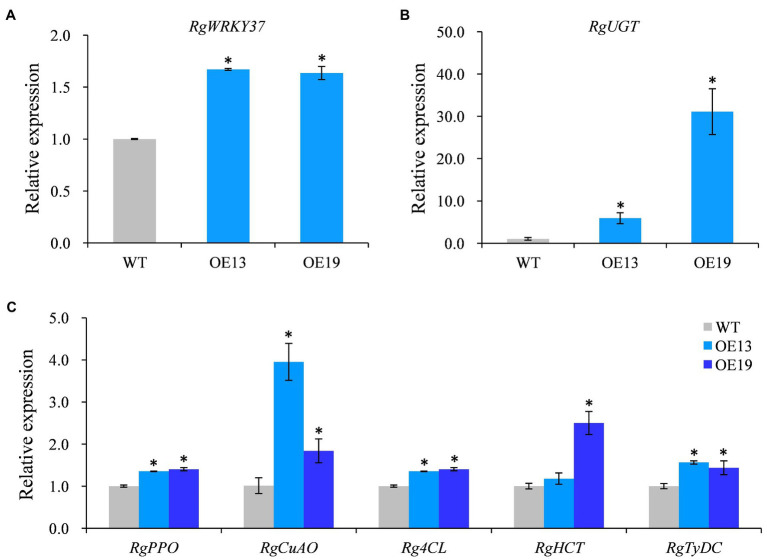
Transcription levels of *RgWRKY37* and the endogenous acteoside biosynthesis genes in hairy roots of transgenic lines. **(A)** Relative expression levels of *RgWRKY37* in transgenic hairy roots. **(B, C)** Relative expression levels of acteoside biosynthetic genes in transgenic hairy roots. The vertical bars show the *SD* values (*n*=3). The asterisks indicate statistically significant differences at *p*<0.05.

To further demonstrate that *RgWRKY37* was positively regulated acteoside biosynthesis, two positive transgenic hairy root lines, lines 13 and 19, were randomly selected for qRT-PCR analysis. The relative expression levels of *RgWRKY37* were found to be significantly increased in lines 13 and 19 compared to the control (Figure 9A). Furthermore, we performed qRT-PCR analysis of the relative expression levels of enzyme genes involved in the acteoside biosynthesis. The results showed that the relative expression of genes encoding UDP-glucose glucosyltransferase (UGT, CL592.Contig1), polyphenol oxidase (PPO), copper-containing amine oxidase (CuAO), 4-coumarate-CoA ligase (4CL), shikimate O-hydroxycinnamoyltransferase (HCT), and tyrosine decarboxylase (TyDC) was significantly upregulated in the two transgenic hairy root lines compared to the wild type (Figures 9B,C).

**Figure 10:**
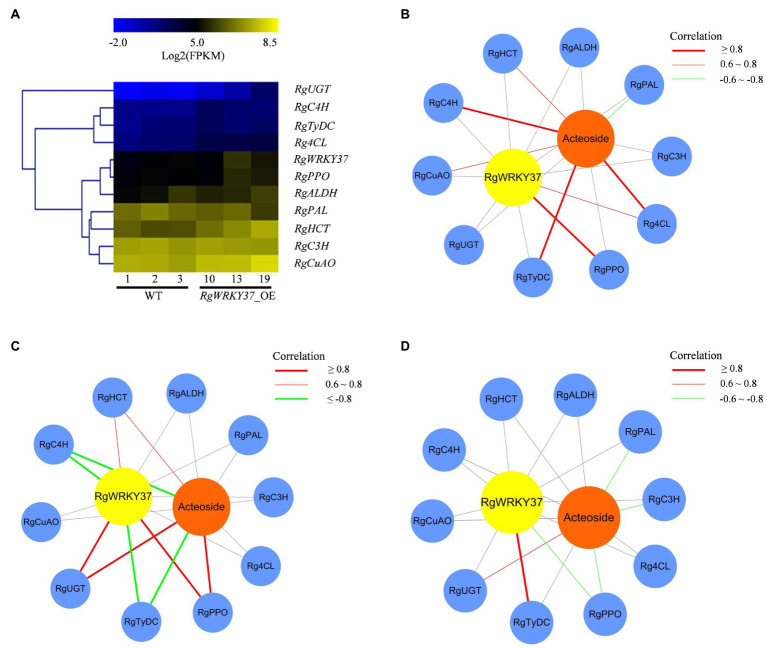
Expression patterns of *RgWRKY37* and enzyme genes involved in acteoside biosynthesis in hairy roots. **(A)** Heat map representing expression dynamics of *RgWRKY37* and candidate enzyme genes in transgenic lines. **(B)** Gene-acteoside correlation network for transgenic hairy root lines. **(C)** Gene-acteoside correlation network for SA-treated hairy roots. **(D)** Gene-acteoside correlation network for H_2_O_2_-treated hairy roots. Genes and acteoside are drawn as blue, yellow, and orange circles, respectively. The red lines indicate positive correlation, the green lines indicate negative correlation, and the gray lines indicate the lack of correlation. Pearson correlation coefficient values were calculated for each gene/metabolite pair.

To determine the relationship among the *RgWRKY37*, enzyme genes, and metabolites, gene expression levels of three transgenic hairy root lines (line 10, 13, and 19) and three wild-type hairy root lines (line 1, 2, and 3) were detected by RNA-seq analysis. Similar to the results of qRT-PCR detections, the expression levels of *RgWRKY37* and eight structural genes (*RgUGT*, *RgC4H*, *RgTyDC*, *Rg4CL*, *RgPPO*, *RgALDH*, *RgHCT*, and *RgCuAO*) were generally higher in transgenic hairy lines than in wild-type lines (Figure 10A; Supplementary Table S10). Pearson’s correlation analysis was conducted between the FPKM values of *RgWRKY37* and enzyme genes and between FPKM values of enzyme genes and acteoside content in transgenic hairy roots and wild-type lines. Gene-metabolite correlation network was constructed for anchoring target genes of *RgWRKY37*. The results showed that *RgTyDC*, *RgC4H*, *Rg4CL*, *RgCuAO*, and *RgHCT* were positively correlated with acteoside, while *RgPPO* and *RgUGT* were weakly correlated with it (Figure 10B). *RgPPO* and *Rg4CL* were coexpressed with *RgWRKY37*, and *RgHCT*, *RgC4H*, *RgCuAO*, *RgUGT*, and *RgTyDC* were weakly correlated with *RgWRKY37* (Figure 10B).

To further investigate the relationship of *RgWRKY37* and acteoside biosynthetic genes in elicitor treatments and in different tissues, three gene-metabolite correlation networks were constructed with acteoside content, FPKM values of *RgWRKY37* and the 10 enzyme genes in SA- and H_2_O_2_-treated *R. glutinosa* hairy roots and 12 tissues, respectively. Three enzyme genes including *RgHCT*, *RgUGT*, and *RgPPO* were positively correlated with acteoside content and *RgWRKY37* levels in SA-treated hairy roots, while *RgTyDC* and *RgC4H* were negatively correlated with them (Figure 10C). The coexpression of *RgHCT*, *RgUGT*, and *RgTyDC* with acteoside content and *RgWRKY37* level in H_2_O_2_-treated hairy roots showed weak positive correlation, while *RgPPO* and *RgC4H* showed weak negative correlation with them (Figure 10D). It is presumed that the mechanism of acteoside accumulation in *R. glutinosa* hairy roots in response to H_2_O_2_ treatment may be different from SA treatment, and it is still unclear how *RgWRKY37* regulate acteoside biosynthesis under SA and H_2_O_2_ treatments. In addition, there was no enzyme gene significantly correlated with *RgWRKY37* in different tissues of *R. glutinosa* plant, and it could be the lower FPKM values of *RgWRKY37* in most of the tissues (Supplementary Figure S6). Based on the above analysis, we concluded that *RgUGT*, *RgHCT*, *RgPPO*, and *RgTyDC* may be the target genes of *RgWRKY37* in acteoside biosynthesis pathway.

The cis-acting element W-box in the promoter of WRKY regulated genes is predominant binding motif for plant WRKYs (Chen et al., 2018). Promoter sequences are necessary to verify whether RgWRKY37 protein activates their transcription of the candidate enzyme genes. However, *R. glutinosa* is lack of a high-quality reference genome, so only partial promoter sequences of *RgUGT*, *RgPPO*, and *RgTyDC* were obtained. Even so, six and two putative W-box sequences were identified in the promoter of *RgHCT* and *RgUGT*, respectively (Supplementary Figure S7), suggesting that RgWRKY37 might directly induce *RgHCT* and *RgUGT* expression.

All these data suggest that *RgWRKY37* is indeed involved in regulating the biosynthesis of acteoside in *R. glutinosa* hairy roots.

### Subcellular Localization of RgWRKY37

**Figure 11:**
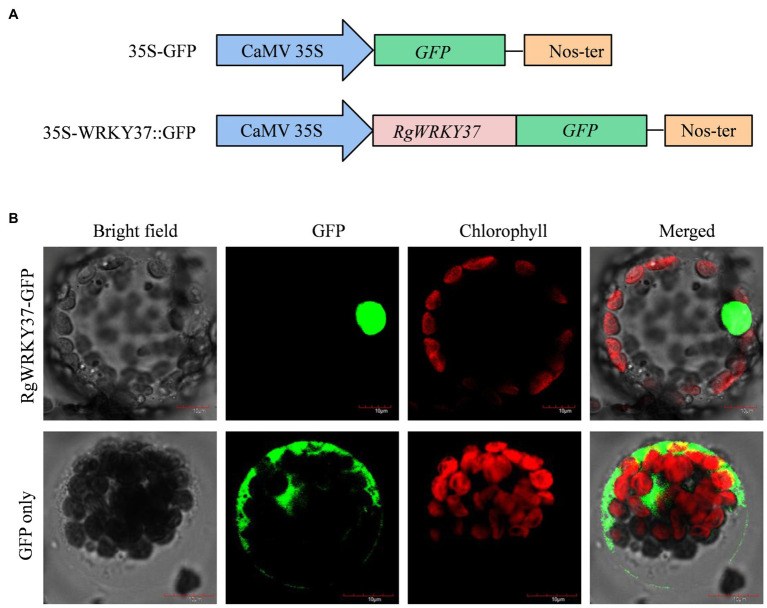
Subcellular localization of the RgWRKY37 protein in tobacco protoplast. **(A)** Schematic diagram of the 35S-RgWRKY37::green fluorescent protein (GFP) fusion protein construct and the 35S-GFP construct. **(B)** Transient expression of the 35S-GFP and the 35S-RgWRKY37::GFP constructs in tobacco protoplast. Green fluorescence corresponding to the expressed proteins was observed with a fluorescence microscope 24h after *Agrobacterium* infection.

The PSORT program was used to predict the subcellular localization of RgWRKY37, and predictions showed that the RgWRKY37 protein contains two putative nuclear localization signals (^93^PAPKDRR^99^ and ^103^KRRK^106^; Supplementary Figure S8). To confirm this prediction, the RgWRKY37 protein was fused to GFP driven by the CaMV 35S promoter to construct a plant expression vector (Figure 11A). Transient transformation of tobacco leaves with the recombinant vector showed that the RgWRKY37-GFP construct was predominantly localized to the nucleus in tobacco protoplasts, while the fluorescence of the GFP construct was presented throughout the cytoplasm and nucleus (Figure 11B). This suggests that RgWRKY37 is located in the nucleus, consistent with its function as a transcription factor.

## Discussion

Although sizes of WRKY proteins vary, all of them contain at least one conserved WRKY domain consisting of a WRKYGQK amino acid sequence and a zinc finger motif (Chen et al., 2018). Initially, the WRKY transcription factors in flowering plants were divided into seven groups (I, IIa, IIb, IIc, IId, IIe, and III) based on the number of WRKY domains and the structure of zinc finger motifs (Eulgem et al., 2000). WRKY proteins with two WRKY domains belong to group I, whereas proteins with one WRKY domain and a C_2_-H_2_ motif belong to group II (Eulgem et al., 2000). In total, 12 and 20 *R. glutinosa* WRKY members belong to the group I and II subfamilies, respectively. WRKY proteins with a single WRKY domain with a C_2_-HC zinc finger motif are distinct from group II WRKY proteins and are assigned to group III (Eulgem et al., 2000; Rinerson et al., 2015). The sequence WRKYGQK in WRKY domains is almost invariant in all of the WRKY proteins (Cheng et al., 2012; Chi et al., 2013). The replacement of any amino acid residues in the WRKYGQK sequence could reduce or eliminate the DNA-binding activity (Maeo et al., 2001). The WRKY domain of RgWRKY27 contains the WRKYGKK sequence, similar to AtWRKY13 and AtWRKY51 (Eulgem et al., 2000), which may have lower DNA-binding activity.

Plants have evolved to develop immunity against a wide variety of microbial pathogens, including resistance (R) protein-mediated immunity against species-specific pathogens and systemic immunity against secondary pathogens (Gao et al., 2011). Two phytohormones, salicylic acid (SA) and jasmonic acid (JA), frequently act antagonistically to mediate defense against specific types of pathogens (Glazebrook, 2005; Koornneef and Pieterse, 2008). An increasing amount of research has indicated that WRKY proteins play important roles in SA- and JA-mediated plant immune responses to various biotic stresses (Chen et al., 2018). AtWRKY25 and AtWRKY23 proteins may interact with MAP kinase 4 substrate 1 (MKS1), which is required for repression of SA-dependent resistance. A *wrky33* knockout mutant was found to exhibit increased expression of the SA-related defense gene *PR1* (Andreasson et al., 2005). AtWRKY50 and AtWRKY51 proteins mediate both SA- and low oleic acid (18:1)-dependent repression of JA signaling, resulting in enhanced resistance to *Alternaria brassicicola* but increased susceptibility to *Botrytis cinerea* (Gao et al., 2011). AtWRKY57 plays a regulatory role in the process of plant immune response and competes with AtWRKY33 in binding to the promoter of JASMONATE ZIM-DOMAIN 1 (JAZ1) and JAZ5 (Jiang and Yu 2016). The majority of *Populus WRKY* genes were induced by both SA and MeJA, and the overexpression of a SA-inducible gene, *PtrWRKY89*, accelerated the expression of PR protein genes and improved resistance to pathogens in transgenic poplar (Jiang et al., 2014a). In *R. glutinosa*, 12 and 10 *RgWRKY* genes were found to be differentially expressed at atleast one of the time points after MeJA and SA treatments, respectively, which suggests that these WRKY genes may participate in immune responses of *R. glutinosa* to biotic stresses.

Senescence is the final stage of leaf development with the programmed cell death process and can be regulated by both endogenous and environmental signals (Guo and Gan, 2005). Phytohormones have been reported to affect leaf senescence *via* complex interconnecting pathways (He et al., 2002). Abscisic acid (ABA), ethylene (ET), SA, jasmonic acid (JA), and brassinosteroids (BR) may promote leaf senescence, while cytokinins and auxin can inhibit leaf senescence (Gan and Amasino, 1997). Both ethylene and SA treatment can promote senescence alone, and SA and ethylene may work together by causing severe early leaf senescence (Wang et al., 2021). Expression pattern analysis revealed that many WRKY genes are strongly induced during senescence, suggesting that WRKY genes are involved in leaf senescence (Guo et al., 2004). Functional analyses reveal that some WRKY genes play important roles in the *Arabidopsis* leaf senescence process. It has been reported that at least 11 WRKY proteins, specifically WRKY22 (Zhou et al., 2011), WRKY54 and WRKY70 (Besseau et al., 2012), WRKY57 (Jiang et al., 2014b), WRKY45 (Chen et al., 2017), WRKY75 (Guo et al., 2017), WRKY6 and WRKY46 (Zhang et al., 2020), WRKY25 and WRKY53 (Doll et al., 2020), and WRKY55 (Wang et al., 2020a), participate in the progression of leaf senescence in *Arabidopsis*. The expression levels of those genes gradually increased during the progression of leaf senescence (Guo et al., 2017; Wang et al., 2020a). In *R. glutinosa*, many *RgWRKY* genes showed a relatively high expression in the old leaf, suggesting these genes may play important roles in *R. glutinosa* leaf senescence.

Acteoside is responsible for the high content of PhGs in *R. glutinosa* and is one of the index components used to measure the quality of *R. glutinosa*. It has multiple pharmacological activities, such as liver protection, anti-inflammatory, antioxidation, and tumor inhibition properties (Wang et al., 2017a). To date, acteoside has been found in more than 200 plant species (Alipieva et al., 2014). However, there are few reports on the causes and molecular functions of acteoside in plants. The isolation of acteoside from an antimicrobial constituent of *Buddleja globosa* leaves has been reported (Pardo et al., 1993). Arciniegas et al. (1997) identified acteoside as an antimicrobial agent that presents antibacterial activity against *Staphylococcus aureus*. Avila et al. (1999) confirmed that acteoside induced a lethal effect on *S. aureus* by affecting protein synthesis and inhibiting leucine incorporation. Moreover, studies have shown that verbascoside isolated from *Plantago major* seeds influenced fungi morphology and decreased sporulation of *Fusarium culmorum*, *Bipolaris sorokiniana*, and *Botrytis cinerea* in barley seedlings (Nikonorova et al., 2009). We accordingly suggest that acteoside may act as an antimicrobial compound in plant defense against certain pathogens.

Acteoside has been detected in both underground (e.g., primary and secondary roots) and aboveground (e.g., stems, leaves, and flowers) parts of plants but at widely varying levels (Alipieva et al., 2014). The concentration of acteoside in the aerial parts of *Verbascum anthophoeniceum* was 0.25%, while in roots of *Sideritis trojana* it was much lower (0.002%; Georgiev et al., 2011; Kirmizibekmez et al., 2012). Among the 12 tissues of *R. glutinosa*, leaves (including L1, L2, and L3) and floral organs (YB, MaB, and MF) were rich in acteoside, but the underground tissues (SS, HTR, and MTR) were lower in acteoside. In recent research, it was found that acteoside content varies in different harvest times of leaves in the same planting year, from 1.71% at September 30 (cultivar BX) up to 5.94% at August 20 (BJ-1; Wang et al., 2017b). SA and MeJA were found to increase the content of acteoside in *Cistanche deserticola* cell cultures (Xu et al., 2005) and *R. glutinosa* hairy roots (Wang et al., 2017a). In this study, we also found that H_2_O_2_ treatment in *R. glutinosa* hairy roots at 24 and 36h effectively improved the acteoside content. It is suggested that the distribution of acteoside is higher in aboveground parts of plants, that acteoside accumulation is easily affected by environmental factors, and that phytohormones are essential signal molecules in regulation of acteoside biosynthesis. These findings will provide important guidance for studying the molecular mechanisms of acteoside biosynthesis.

The significance of WRKY TFs in the regulation of specialized metabolism has been reported in several plant species. For example, *OsWRKY45* is required for priming of diterpenoid phytoalexin biosynthesis after *Magnaporthe oryzae* inoculation (Akagi et al., 2014). AtWRKY18 and AtWRKY40 are implicated in camalexin and indole-glucosinolate biosynthesis, and the accumulation of these compounds was required for resistance toward *Golovinomyces orontii* (Schön et al., 2013). The ectopic expression of *AtWRKY18* and *AtWRK40* together with *AtMYC2* activated the MEP (2-C-methyl-D-erythritol 4-phosphate) pathway in *Salvia sclarea* hairy roots and consequently increased the content of abietane diterpene (Alfieri et al., 2018). CrWRKY1 positively regulates several key TIA pathway genes, especially the TDC gene, resulting in an increase in tryptamine followed by serpentine accumulation (Suttipanta et al., 2011). In this study, we analyzed the relationship of acteoside accumulation and expression levels of *RgWRKY* genes and specified five genes, namely, *RgWRKY7*, *RgWRKY23*, *RgWRKY34*, *RgWRKY35*, and *RgWRKY37*, as candidate regulators for acteoside biosynthesis. In addition, the overexpression of *RgWRKY37* upregulated *UGT* (CL592.Contig1, encoding a UGT83A1 member), *RgPPO*, *RgCuAO*, *Rg4CL*, *RgHCT*, and *RgTyDC*, resulting in an increase in the content of acteoside and total PhGs in *R. glutinosa* hairy roots.

Construct gene-to-metabolite network has become an effective method to screen the transcription factor and their target genes in compounds biosynthetic pathway in plants. Using MicroTom Metabolic Network, researchers systematically studied the major metabolic changes that occur throughout the tomato growth cycle and identified novel transcription factors that regulate the biosynthesis of important secondary metabolites (Li et al., 2020b). Through applying transcript-metabolite correlation analysis, researchers construct the regulatory network of *IiWRKY34* for lignan biosynthesis and identified a total of 11 pathway genes that were correlated with *IiWRKY34* and at least one lignan (Xiao et al., 2020). Here, gene-metabolite correlation regulatory networks of *RgWRKY37* for acteoside biosynthesis were constructed with the abundances of acteoside biosynthetic genes and acteoside. The results showed that several enzyme genes were correlated with *RgWRKY37* and acteoside, but the identified gene/metabolite pairs were inconsistent in different experiments. For example, *RgPPO* was strongly coexpressed with *RgWRKY37* both in transgenic hairy roots and in SA-treated hairy roots, but it weakly correlated with *RgWRKY37* in H_2_O_2_-treated hairy roots. *RgTyDC* was positively and negatively correlated with *RgWRKY37* in SA- and H_2_O_2_-treated hairy roots, respectively. It is suggested that the mechanism of *RgWRKY37* regulate metabolism under SA and H_2_O_2_ treatments was different. A previous study showed that overexpression of the tyrosine decarboxylase gene *MdTyDC* in apple induced higher dopamine levels and enhanced antioxidant enzyme activities and gene expression, resulting decreased the accumulation of H_2_O_2_ (Wang et al., 2020b). It is probably that exogenous application of H_2_O_2_ induced *RgWRKY37* expression and then increased dopamine content by activated the expression of *RgTyDC* to enhance H_2_O_2_ scavenging. SA may directly inactivate catalases (CATs) and ascorbate peroxidases (APXs), thus leading to H_2_O_2_ accumulation (Rao et al., 1997). In *R. glutinosa* hairy roots, exogenous application of SA may lead to higher H_2_O_2_ concentration, indicate that H_2_O_2_ scavenging was repressed, which consistent with the decreased expression of *RgTyDC* in SA-treated hairy roots. It is suggested that both SA and H_2_O_2_ could improve acteoside accumulation, but with different mechanisms.

## Conclusion

In this research, we identified 37 WRKY genes in *R. glutinosa* transcriptome and analyzed their expression patterns in response to SA, MeJA, and H_2_O_2_ treatments and their expression profiles in 12 tissues of *R. glutinosa* plants. Combined with changes in acteoside accumulation, we screened *RgWRKY7*, *RgWRKY23*, *RgWRKY34*, *RgWRKY35*, and *RgWRKY37* as candidate transcript factors in regulating acteoside biosynthesis. Overexpressed of *RgWRKY37* in *R. glutinosa* hairy roots caused increasing of acteoside content, suggest that *RgWRKY37* is a regulator in acteoside biosynthesis.

## Data Availability Statement

The data sets presented in this study can be found in online repositories. The names of the repository/repositories and accession number(s) can be found at: NCBI SRA Bioproject, accession no: PRJNA382479; Genome Sequence Archive, accession numbers: PRJCA006052, PRJCA006054, and PRJCA006055.

## Author Contributions

FW conceived the project and designed the experiments. FW, XL, XZ, ML, CM, JZ, YL, and XY performed experiments. FW, XL, and XZ prepared the manuscript together. CX and XL participated in data analysis and revised the manuscript. All authors contributed to the article and approved the submitted version.

## Funding

This project was supported by the National Science Foundation of China (NSFC Grant No. 81872950, 82073952, and 81473299).

## Conflict of Interest

The authors declare that the research was conducted in the absence of any commercial or financial relationships that could be construed as a potential conflict of interest.

## Publisher’s Note

All claims expressed in this article are solely those of the authors and do not necessarily represent those of their affiliated organizations, or those of the publisher, the editors and the reviewers. Any product that may be evaluated in this article, or claim that may be made by its manufacturer, is not guaranteed or endorsed by the publisher.

## Footnotes

^1^ http://www.ncbi.nlm.nih.gov/gorf/orfig.cgi

^2^ https://ngdc.cncb.ac.cn/gsa/

^3^ https://www.ncbi.nlm.nih.gov/tools/primer-blast/

^4^ http://www.ncbi.nlm.nih.gov/nuccore

^5^ http://www.ncbi.nlm.nih.gov/gorf/gorf.html
