# Supplementary material for: Transcriptome-Wide Identification of WRKY Transcription Factor and Functional Characterization of RgWRKY37 Involved in Acteoside Biosynthesis in Rehmannia glutinosa
Source: Front Plant Sci. 2021 Sep 29;12:739853. doi: 10.3389/fpls.2021.739853 (PMC8511629; doi:10.3389/fpls.2021.739853)
Supplement: Supplementary file 1 [file Data_Sheet_1.docx]

Supplementary Material

Transcriptome-wide identification of WRKY transcription factor and functional characterization of *RgWRKY37* involved in acteoside biosynthesis in *Rehmannia glutinosa*

Fengqing Wang^1^*, Xinrong Li^1^, Xin Zuo^1^, Mingming Li^1^, Chunyan Miao^1^, Jingyu Zhi^1^, Yajing Li^1^, Xu Yang^1^, Xiangyang Liu^1^, Caixia Xie^2^

*** Correspondence:** Corresponding Author: fqwang@henau.edu.cn

Supplementary Figures


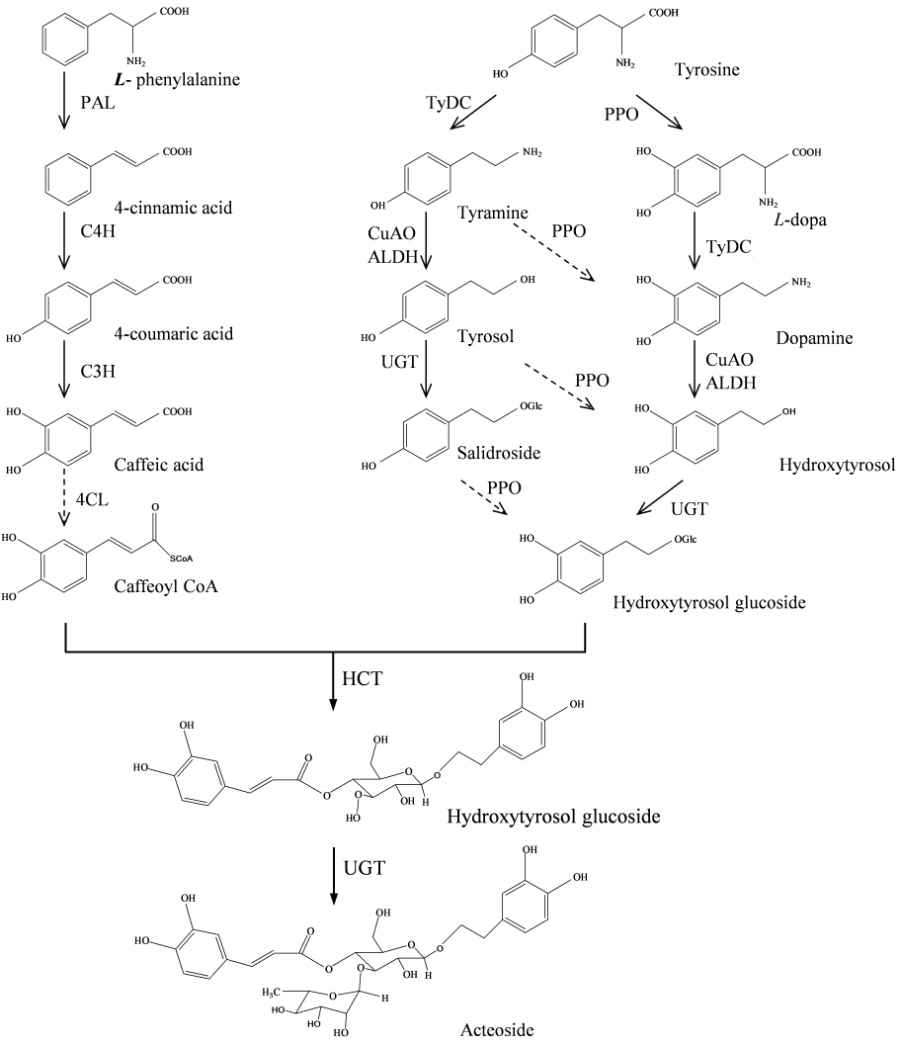


**FIGURE S1. The putative acteoside biosynthesis pathway.** PAL: phenylalanine ammonia-lyase; C4H, cinnamate-4-hydroxylase; C3H, coumarate-3-hydroxylase; TyDC, tyrosine decarboxylase; PPO, polyphenol oxidase; CuAO, copper-containing amine oxidase; ALDH, alcohol dehydrogenase; UGT, UDP-glucose glucosyltransferase; 4CL, 4-coumarate-CoA ligase; HCT, Shikimate O-hydroxycinnamoyltransferase.


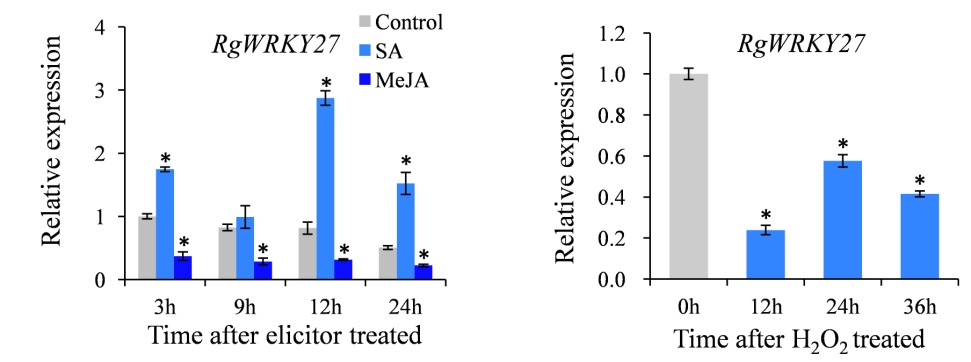


**FIGURE S2. Relative expression levels of *RgWRKY27* in *R. glutinosa* hairy roots under SA, MeJA and H_2_O_2_ treatments.** Relative expression levels were determined by Real-time PCR (qRT-PCR). Vertical bars indicate the standard deviation of three biological replicates. Asterisks indicate a significant difference at the *p* < 0.05 level.


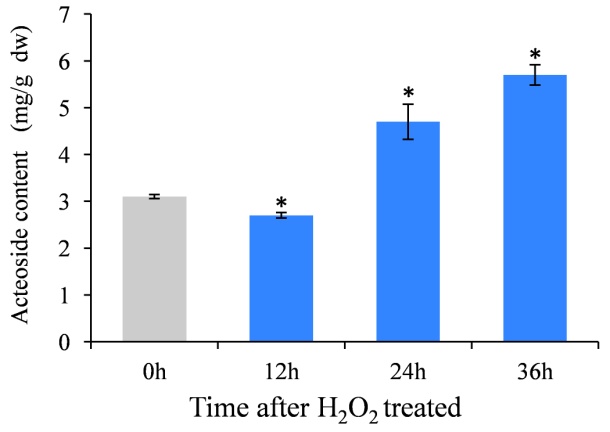


**FIGURE S3. Acteoside content in *R. glutinosa* hairy roots under H_2_O_2_ treatment.** Vertical bars indicate the standard deviation of three biological replicates. Asterisks indicate a significant difference at the *p* < 0.05 level.


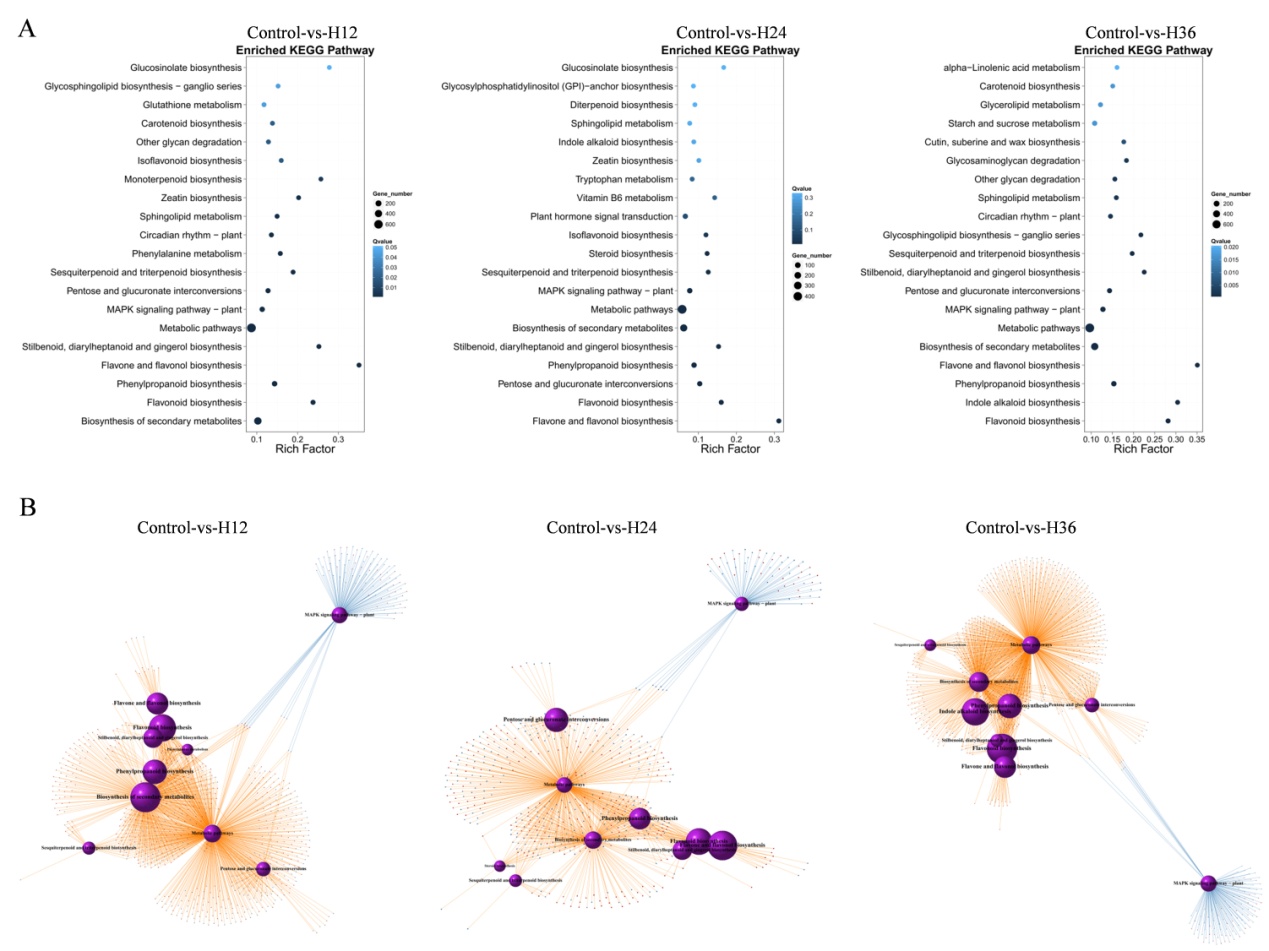


**FIGURE S4. Top 20 enriched KEGG pathways among the annotated DGEs across three comparisons. (A)** Top 20 enriched KEGG pathways in H_2_O_2_ treatment comparisons. The Y-axis on the left represents KEGG pathways, and the X-axis indicates the enrichment factor. Low q-values are shown in blue, and high q-values are depicted in dark blue. **(B)** Gene regulatory network of co-enriched DGEs in KEGG pathways across the three comparisons.


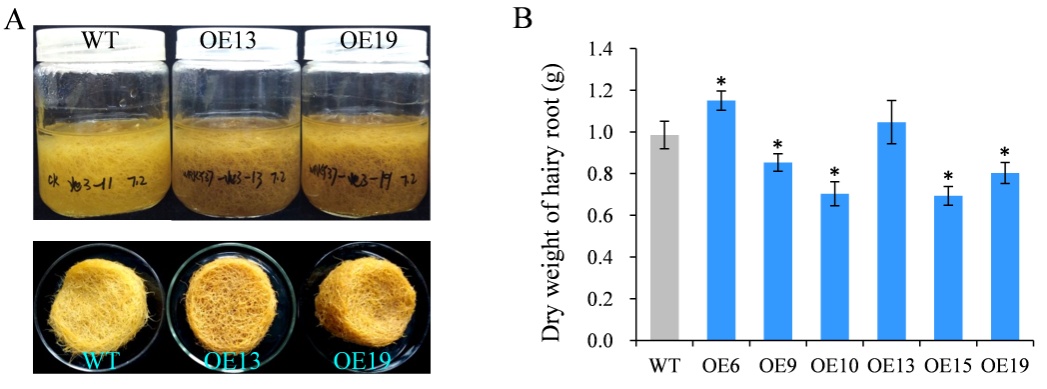


**FIGURE S5. Phenotype of transgenic hairy roots of *R. glutinosa.*** **(A)** Phenotypes of *RgWRKY37* over-expressed and wild type (WT) hairy roots. **(B)** Dry weight of transgenic hairy roots after cultured in liquid MS medium for 45 days. The vertical bars show the SD values (n=3). The asterisks indicate statistically significant differences at *p* < 0.05.


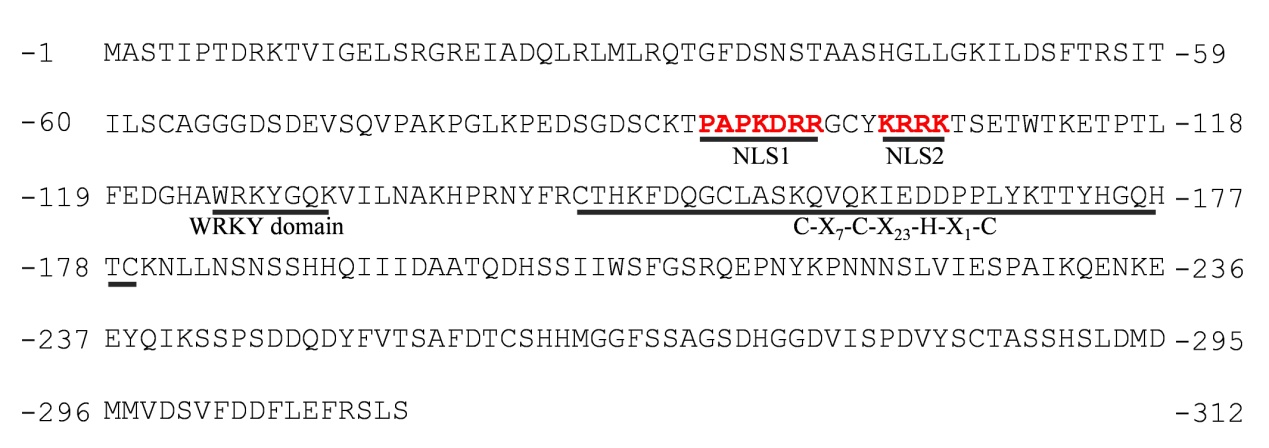


**FIGURE S6. Protein sequence of RgWRKY37.** NSL, nuclear *localization* signal.


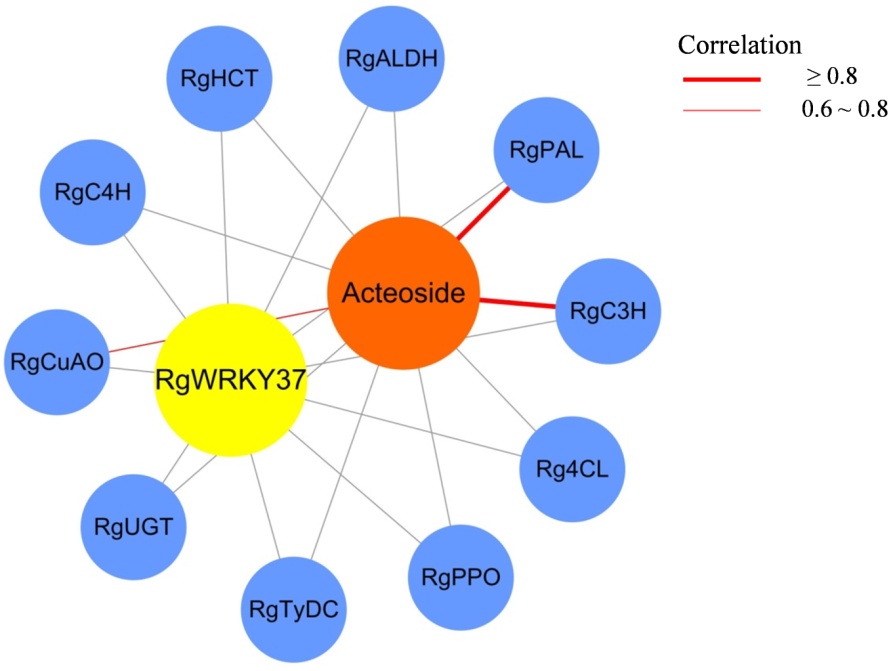


**FIGURE S7. Gene-acteoside correlation network for twelve tissues of *R. glutinosa***. Genes and acteoside are drawn as blue, yellow and orange circles, respectively. The red lines indicate positive correlation, and the gray lines indicate the lack of correlation. The numbers indicate correlation coefficient.

**
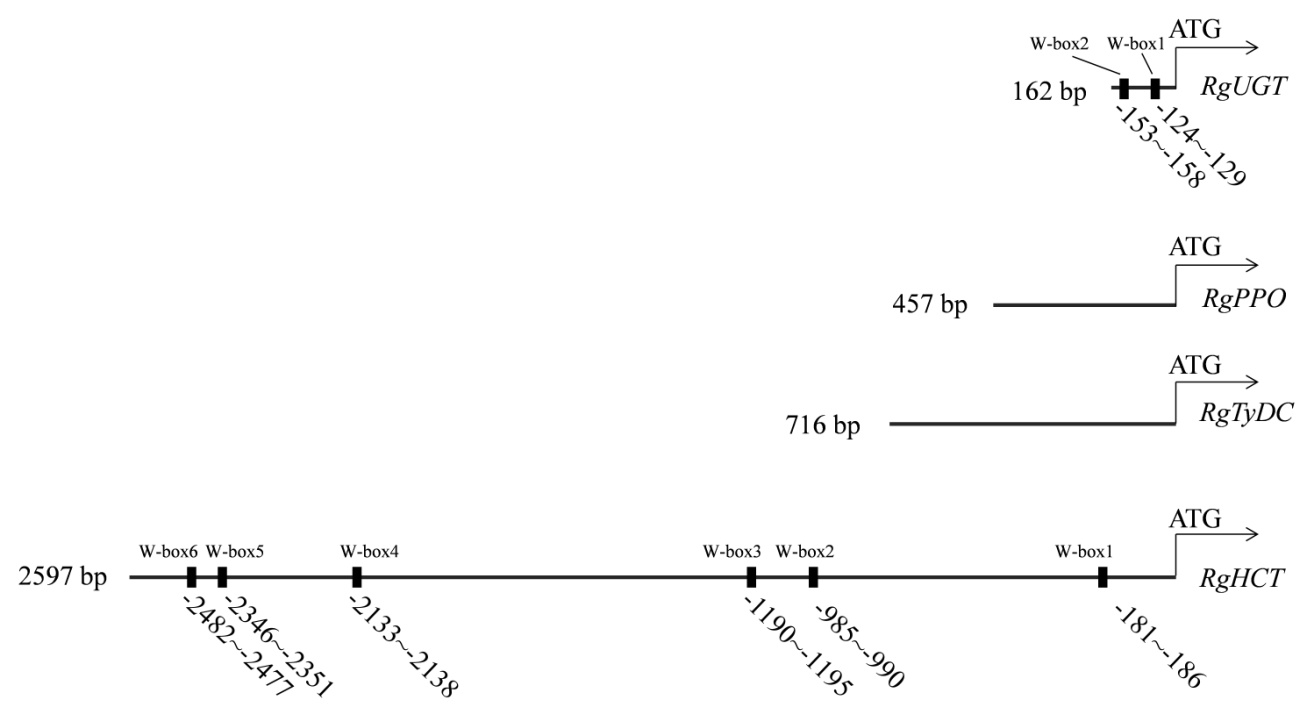
**

**FIGURE S8. Schematic diagram indicating the locations of putative RgWRKY37 binding sites in the promoters of the candidate enzyme genes.**
